# Immunotherapy with subcutaneous immunogenic autologous tumor lysate increases murine glioblastoma survival

**DOI:** 10.1038/s41598-017-12584-0

**Published:** 2017-10-24

**Authors:** Jochen Belmans, Matthias Van Woensel, Brecht Creyns, Joost Dejaegher, Dominique M. Bullens, Stefaan W. Van Gool

**Affiliations:** 1 Laboratory of Pediatric Immunology, KU Leuven, Herestraat 49 box 811, Leuven, 3000 Belgium; 2 Research Group Experimental Neurosurgery and Neuroanatomy, KU Leuven, Herestraat 49, Leuven, 3000 Belgium; 3 Laboratoire de Pharmacie Galénique et de Biopharmacie, Université libre de Bruxelles (ULB), Boulevard du triomphe CP207, Brussels, 1050 Belgium; 4 Laboratory of Clinical Immunology, KU Leuven, Herestraat 49 box 811, Leuven, 3000 Belgium; 5 Division of Clinical Pediatrics, University Hospitals Leuven, Herestraat 49, Leuven, 3000 Belgium; 6 Medizinische Leitung der Translationalen Onkologie, Immunologisches Onkologisches Zentrum, Köln, Germany

## Abstract

Immunotherapeutic strategies for glioblastoma, the most frequent malignant primary brain tumor, aim to improve its disastrous consequences. On top of the standard treatment, one strategy uses T cell activation by autologous dendritic cells (DC) *ex vivo* loaded with tumor lysate to attack remaining cancer cells. Wondering whether ‘targeting’ *in vivo* DCs could replace these *ex vivo* ones, immunogenic autologous tumor lysate was used to treat glioma-inoculated mice in the absence of *ex vivo* loaded DCs. Potential immune mechanisms were studied in two orthotopic, immunocompetent murine glioma models. Pre-tumoral subcutaneous lysate treatment resulted in a survival benefit comparable to subcutaneous DC therapy. Focussing on the immune response, glioma T cell infiltration was observed in parallel with decreased amounts of regulatory T cells. Moreover, these results were accompanied by the presence of strong tumor-specific immunological memory, shown by complete survival of a second glioblastoma tumor, inoculated 100 days after the first one. Finally, in combination with temozolomide, survival of established glioma in mice could be increased. Our results show the potential of immunogenic autologous tumor lysate used to treat murine glioblastoma, which will be worthwhile to study in clinical trials as it has potential as a cost-efficient adjuvant treatment strategy for gliomas.

## Introduction

Amongst brain tumors, glioblastoma multiforme (GBM) can be considered the greatest nemesis, being the most aggressive and common primary brain tumor^1^. The disease is part of the high grade gliomas (HGG) and is classified as a grade IV malignancy according to the World Health Organisation^2,3^. With a limited incidence of 4 in 100.000 people, GBM still has a very high socioeconomic impact^4,5^. Even though patients are intensively treated, the prognosis remains dismal with a median survival of only 14.6 months^6,7^. Therefore a lot of effort is put in adjuvant therapies such as cancer immunotherapy^8,9^.

For GBM, especially vaccination strategies using dendritic cells (DC) have been tested, frequently with the use of whole tumor lysate^10^. DC immunotherapy consists of patient-derived DCs differentiated *ex vivo* often from harvested monocytes and loaded with autologous tumor lysate. Recently a meta-analysis shows that DC immunotherapy has a limited long-term effect in human GBM^11^. Within our group preclinical optimisation of DC immunotherapy in the murine GL261 glioma model has been studied^12,13^. Therefore, different formulations of tumor lysate were created and tested *in vitro* and *in vivo*. Both cited studies conclude that the immunogenicity of the lysate loaded to the DCs is important. Changing the protocol to create lysate will change its immunogenicity.

The effect of tumor lysate without the addition of DCs to treat cancer has been reported for different cancer types e.g. melanoma, prostate cancer, glioma, …^14–16^. Studies describing this effect often make use of adjuvants added to the lysate in different forms e.g. cytokines such as GM-CSF and IL12^17^; (gene transduced) allogenic cells^15,18–20^, heat shock proteins (HSP65 and HSP96)^16,21^ or CpG oligodeoxynucleotide^22,23^. Injection of autologous tumor lysate in the absence of adjuvants was performed in only two studies as a cancer treatment^24–26^. However some remarks need to be taken into account. In a preclinical glioma model by Jouanneau *et al*., an impressive improvement in long-term survival was observed in glioma-bearing mice treated with tumor lysate^24^. Tumor lysate in that study was used as a boost injection after initial priming with lysate loaded DCs. These boost injections were superior when compared to a second lysate loaded DC vaccination in their model. In a human clinical trial, to treat renal cell carcinoma, autologous lysate treatment was used as an adjuvant therapy after nephrectomy^25,26^. Before lysate preparation, isolated tumor cells were grown in medium supplemented with interferon-γ (IFN-γ) and tocopherol acetate to increase their immunogenicity. Autologous tumor cell vaccination in comparison to no additional treatment resulted in an improved survival in T3 staged patients. These studies show that injecting lysate has potential as a treatment modality.

Here we investigated the potential effect and immune mechanisms of immunogenic autologous tumor lysate, in the absence of DCs and adjuvants, to treat malignant gliomas in an orthotopic mouse model. We hypothesize that lysate once injected subcutaneously will be taken up by skin residing DCs *in vivo* and this way is able to create an immune response against tumor cells. Our findings suggest autologous lysate treatment is able to create an immune response with long-lasting memory in a murine glioma model. As the injection of tumor lysate instead of *ex vivo* loaded DCs is more cost efficient and reduces the number of procedures by personnel, the described study is justified and might have important implications in how to “vaccinate” GBM patients in combination with standard therapy.

## Results

### Uptake of lysate and presentation of lysate fragments by DCs *in vitro*

**Figure 1:**
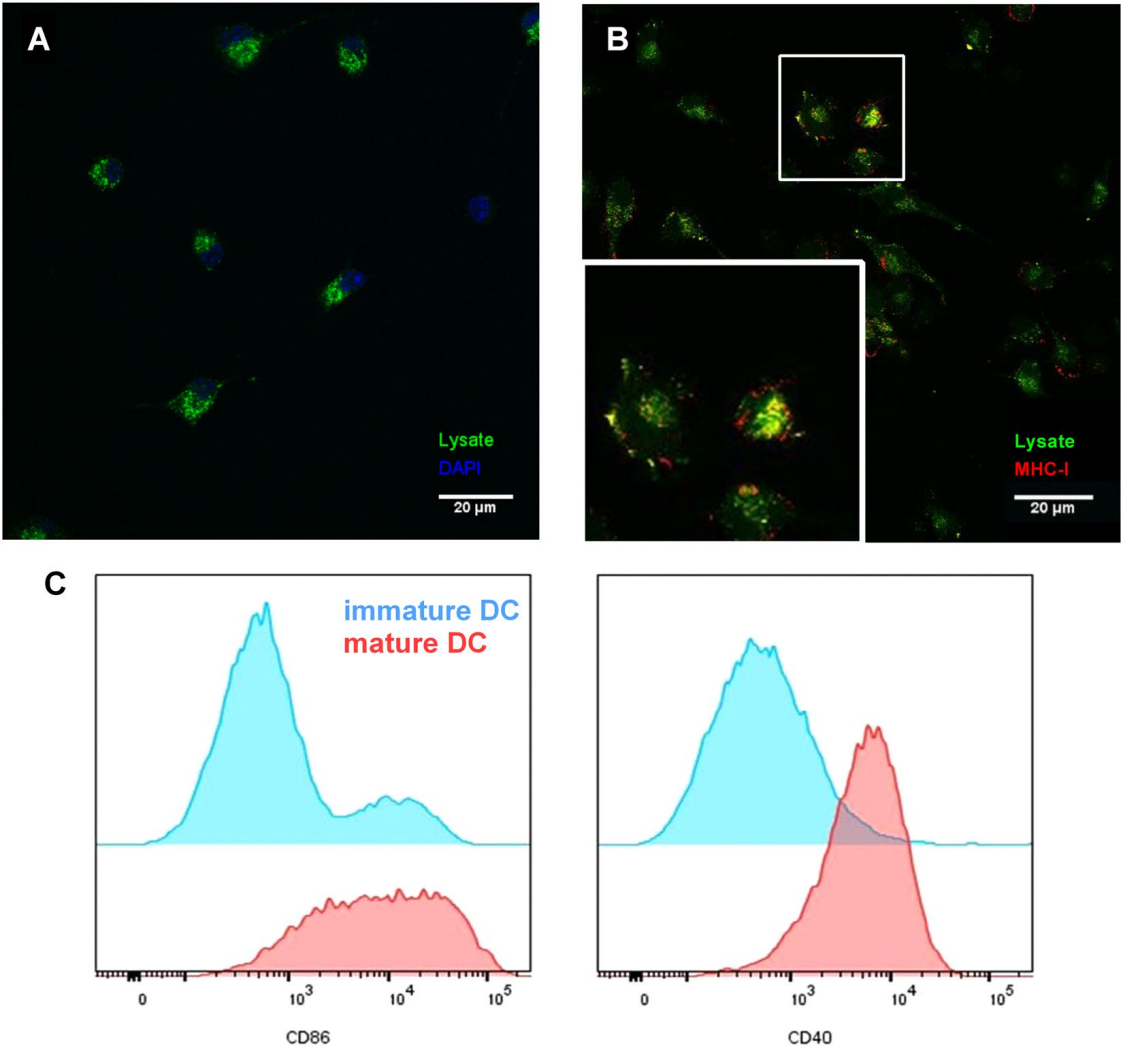
Uptake and cross-presentation of lysate fragments by dendritic cells *in vitro*. *In vitro* differentiated DCs were incubated with FITC labelled lysate for 90 minutes, afterwards washed and stained in the uptake experiments **(A)** or matured with LPS in the cross-presentation experiments **(B)**. **(A)** Z-stack images, obtained with confocal microscopy, were used to visualize DCs and prove real uptake of lysate. Picture **(A)** represents one slice of the confocal z-stack with DAPI nucleus staining (blue) and FITC signal (green). **(B)** Maturation of DCs was ended after 24 hours by washing cells, fixation and staining. **(B)** Confocal microscopy showed colocalization (yellow signal) of fragments of lysate (FITC, green signal) and MHC class I molecules (PE, red signal). The box at the bottom is a magnification of the marked cells. **(C)** Flow cytometry was used to study the expression of maturation markers on lysate loaded DCs. *In vitro* differentiated DCs were incubated with lysate for 90 minutes, washed and after 24 hours incubation stained. Representative graphs for DC maturation markers CD40 and CD86 are shown with expression on immature DCs in blue and marker expression by mature DCs in red. Images were visualized with a 400x magnification and all pictures and graphs are representative of at least three independent experiments.

Before *in vivo* testing, we investigated uptake of tumor lysate fragments by DCs *in vitro* as previously published by De Vleeschouwer *et al*. in a human setting^27^. For this, lysate was labelled with FITC and added *in vitro* to iDCs. Both fluorescence microscopy and flow cytometry indicated the uptake of lysate fragments by visualisation of FITC positive DCs (data not shown). To discriminate between uptake of lysate *versus* surface adhesion to DC membranes, confocal microscopy was performed. We could demonstrate that lysate-FITC complexes were internalized after 90 minutes of incubation (Fig. 1A and Supplementary movie). Confirmation of this observation was obtained by trypan blue quenching of the surface FITC signal (Supplementary Fig. 1, upper panel). Following FITC surface quenching the positive intracellular signal was still present, indicating uptake by DCs. Moreover, to examine whether the uptake is an active physiologic process, loading of DCs with lysate-FITC was performed at 37 °C or at 4 °C, a temperature at which all cell metabolism is shut down^27^. Uptake of lysate fragments was only observed in the 37 °C condition, indicating an active uptake mechanism of these fragments by DCs (Supplementary Fig. 1, lower panel).

To complete the *in vitro* study we aimed to demonstrate the processing of lysate, which is necessary to create an immune response against tumor cells. Using confocal microscopy, colocalization of FITC-lysate fragments with MHC-I molecules was found (Fig. 1B). Moreover, maturation of DCs was observed when lysate was presented to the DCs (Fig. 1C and Supplementary Fig. 2). Together these results indicate that (cross-)presentation of lysate fragments in MHC-I context is possible when lysate is loaded to DCs.

### Pre-tumor lysate treatment improves survival in a murine glioma model

**Figure 2:**
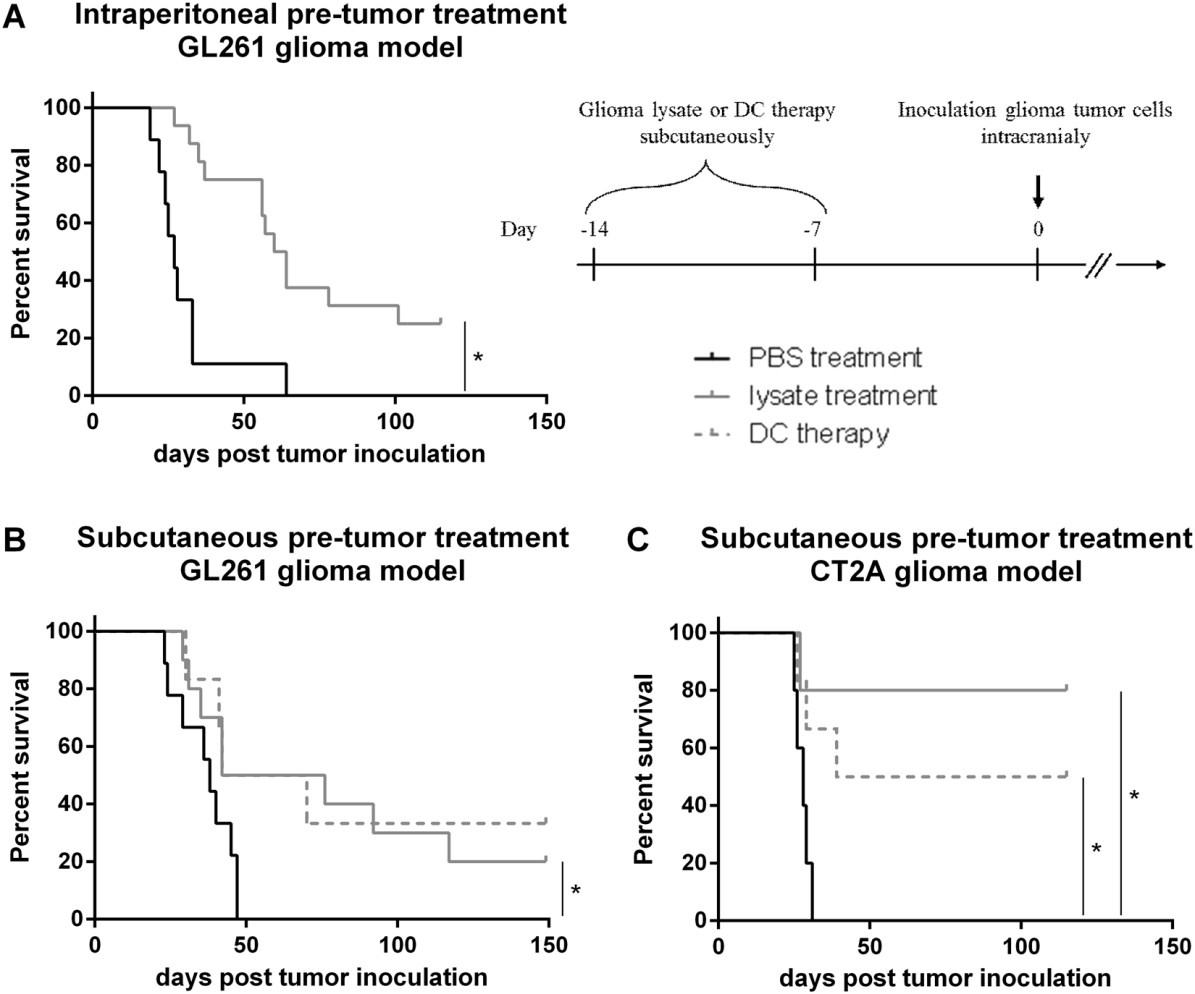
Pre-tumor lysate treatment results in similar survival improvement as compared to dendritic cell therapy in 2 glioma mouse models. Mice were treated with autologous lysate 14 and 7 days before tumor inoculation with 5 × 10^5^ glioma cells. In the GL261 glioma model both **(A)** intraperitoneal and **(B)** subcutaneous injection of lysate (n = 16 and n = 10 respectively) were studied in comparison to PBS treatment (n = 9). **(C)** For the CT2A glioma model only subcutaneous lysate treatment was tested. Moreover, in the two bottom graphs (subcutaneous injection), dendritic cell therapy (n = 6) was performed and applied as a literature based control condition. For graphs **(A)** and **(B)** data of two independent experiments were pooled. Statistical significance was calculated by Log-rank test, *p < 0.05.

Following the *in vitro* confirmation of lysate uptake and processing by DCs, we wanted to study whether autologous lysate is clinically active. In an intracranial GL261 glioma model of fully immunocompetent mice we compared tumor growth in the absence or presence of treatment. The murine GL261 glioma model is the standard model to test immunotherapy in GBM and has already been used in our group for over a decade^28–30^. Due to the aggressive nature of GL261 glioma, prophylactic immunisation was performed 14 and 7 days before tumor inoculation (Fig. 2). In line with previous published studies, we started testing the clinical activity of lysate after i.p. injection^31^. In comparison to mock treated mice, animals in the test-condition showed an increased median survival with 25% LT-surviving mice (Fig. 2A). With the relative amount of DCs being greater in the skin as compared to the peritoneum^32^ and our hypothesis stating these cells as the most important cells to create an immune response following lysate injection, the s.c. injection route was studied next. As observed after i.p. injections, s.c. pre-tumor lysate treatment showed a similar positive effect on the survival of mice in comparison to mock injected mice (Fig. 2B). By including a group of mice treated with s.c. DC therapy, no differences could be observed in comparison to lysate treatment. We then studied whether we could confirm these results in the CT2A cell line, predominantly used for studying glioma stem cells, but with several GBM features such as a high infiltrative nature and high mitotic index and cell density^30,33^. Retaining the pre-tumor treatment schedule of the GL261 model, the CT2A model resulted in 80% of the lysate-treated mice surviving tumor inoculation over 120 days (Fig. 2C). In comparison, the s.c. DC therapy in this CT2A model also improved survival, although less effective as the lysate treatment. These results clearly indicate the potential of autologous lysate therapy in glioma mouse models.

In search for a more universal immunotherapy treatment, some researchers have suggested to use the lysate of multiple HLA-matching reference tumor cell lines^18^. Therefore we wondered whether a non-autologous treatment would be able to treat mice glioma. This can be elicited by pre-treatment of mice with lysate of one cell line and testing if this could help mice intracranially injected with tumor cells of the other cell line. GL261 and CT2A cells are both syngeneic cell lines for C57BL/6 mice but originate from different cells^30^ and therefore could be used to test the effect of syngeneic treatment with non-autologous tumor lysate (Supplementary Fig. 3). Syngeneic pre-tumor treatment with CT2A lysate resulted in increased survival of GL261 cell inoculated mice, with three out of four mice surviving tumor over 150 days. Syngeneic pre-tumor treatment with GL261 lysate however did not show significant improvement for CT2A glioma-bearing mice, although one mouse did survive tumor inoculation over 100 days. Showing one-way cross-reactivity, these results suggest the importance of (tumor) specificity in GBM.

### Early after tumor inoculation, lysate treatment induced a T cell influx combined with diminished immune suppression in the brain

**Figure 3:**
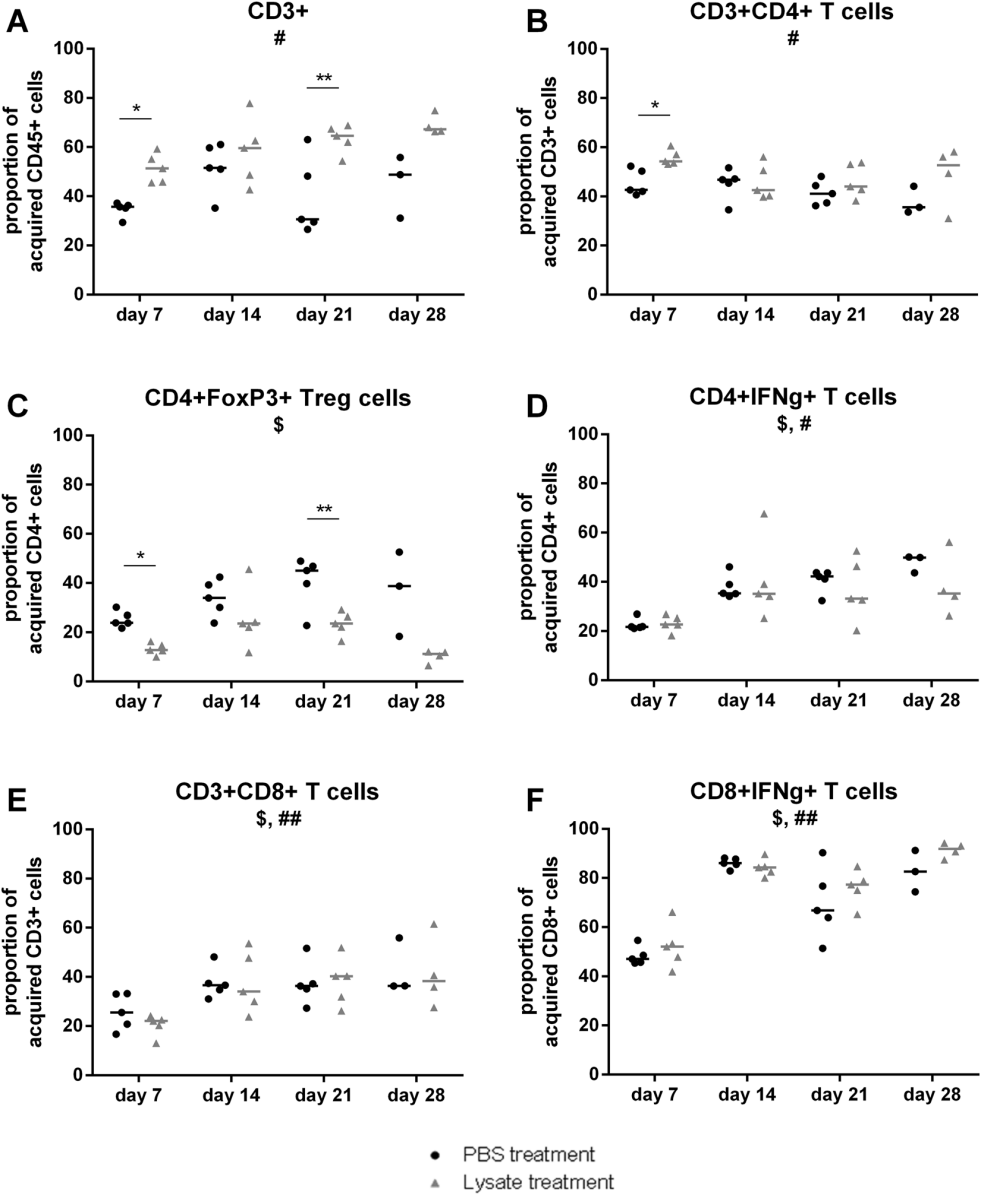
Early after tumor inoculation, lysate treatment induced a T cell influx combined with diminished immune suppression in the brain. Brain immune contexture of pre-tumor treated mice was studied 7, 14, 21 and 28 days after tumor inoculation. Graphs present kinetic analyses of different T cell population proportions in PBS treated (●) or lysate treated (∆) mice. Cell populations were defined by different stainings with **(A)** CD3^+^ lymphocytes as single cells, ZY-, CD45+, CD3+ gated to CD45+; **(B)** CD4^+^ T cells as single cells, ZY-, CD45+, CD3+, CD4+ gated to CD3+; **(C)** Tregs as single cells, ZY-, CD45+, CD3+, CD4+, FoxP3+ gated to CD4+; **(D)** IFNγ-producing CD4^+^ T cells as single cells, ZY-, CD45+, CD3+, CD4+, IFNγ+ gated to CD4+; **(E)** CD8^+^ T cells as single cells, ZY-, CD45+, CD3+,CD8+ gated to CD3+; **(F)** IFNγ-producing CD8^+^ T cells as single cells, ZY-, CD45+, CD3+,CD8+, IFNγ+ gated to CD8+. Statistical significance was calculated by two-way ANOVA. Groups of mice consisted of 5 mice except for day 28, when 1 or 2 mice already died due to glioma growth in the lysate and PBS treated conditions respectively. Significant differences between two treatment conditions, PBS or lysate, are indicated by asterisks: *p < 0.05; **p < 0.01. The symbols $ and # below the graph title indicate significant changes over time within the PBS treated and lysate treated populations respectively. $ or ^#^p < 0.05; ^##^p < 0.01.

To investigate the immune response induced by autologous lysate treatment, brain infiltrating immune cells, and more specifically the T cell populations, were studied. Because of its invasive character, more resembling GBM, the CT2A model was used for this experiment^30,33^. Pre-treated mice were tumor inoculated and sacrificed 7, 14, 21 or 28 days later to isolate the brain immune cells. Flow cytometry was used to study the proportions of different T cell populations of lysate treated mice in comparison to mock treated animals. The gating strategy used for the population analysis is depicted in Supplementary Figs 4 and 5. At the earliest time point, day 7, influx of total T lymphocytes (CD3^+^) was detected. As shown in Fig. 3, this resulted in an increased proportion of CD4^+^ but not CD8^+^ T cells. Although the relative amount of CD4^+^ T cells was increased, the proportion of CD4^+^Foxp3^+^ regulatory T cells (Treg) was decreased, pointing to decreased immune suppression. Together these results indicate a shift from immune suppression to immune activation 7 days after tumor inoculation due to lysate pre-treatment. At day 21, but not day 14 nor day 28, we also observed an increased proportion of total T lymphocytes, with a lower frequency of Tregs infiltrating the tumor environment (Fig. 3).

From a longitudinal point of view (Fig. 3), an increased proportion of CD3^+^ T cells was observed which correlated with increased relative amount of CD8^+^ T cells. Importantly, this increase of CD8^+^ T cells was accompanied by an influx of IFNγ-producing CD8^+^ T cells over time both for the lysate treated and PBS treated animals. Kinetic analysis of CD4^+^ T cells showed a decrease in frequency starting between day 7 and 14, indicating an important role mainly at the first time point. Overall it was concluded that the immune response is driven both by CD4^+^ and CD8^+^ T cells.

Peripheral immune response was studied by investigating T cell populations in spleen and draining lymph nodes of pre-treated mice at day 21 after tumor inoculation. For the draining lymph nodes, cells from inguinal and axillary lymph nodes were pooled and the same flow cytometric stainings and gating strategy as for the brain infiltrating immune cell study were used. Among the splenocytes, a decreased proportion in Treg cells was detected for autologous lysate treated mice as compared to PBS treated animals (data not shown). On the other hand, no difference was detected for the IFN-γ producing T cells. Moreover, neither cell population showed any difference in the lymph nodes. It can be concluded that these data suggest the presence of an overall immune-suppression by autologous lysate treatment.

### Pre-tumor lysate treatment induces a long-lasting immunological memory

**Figure 4:**
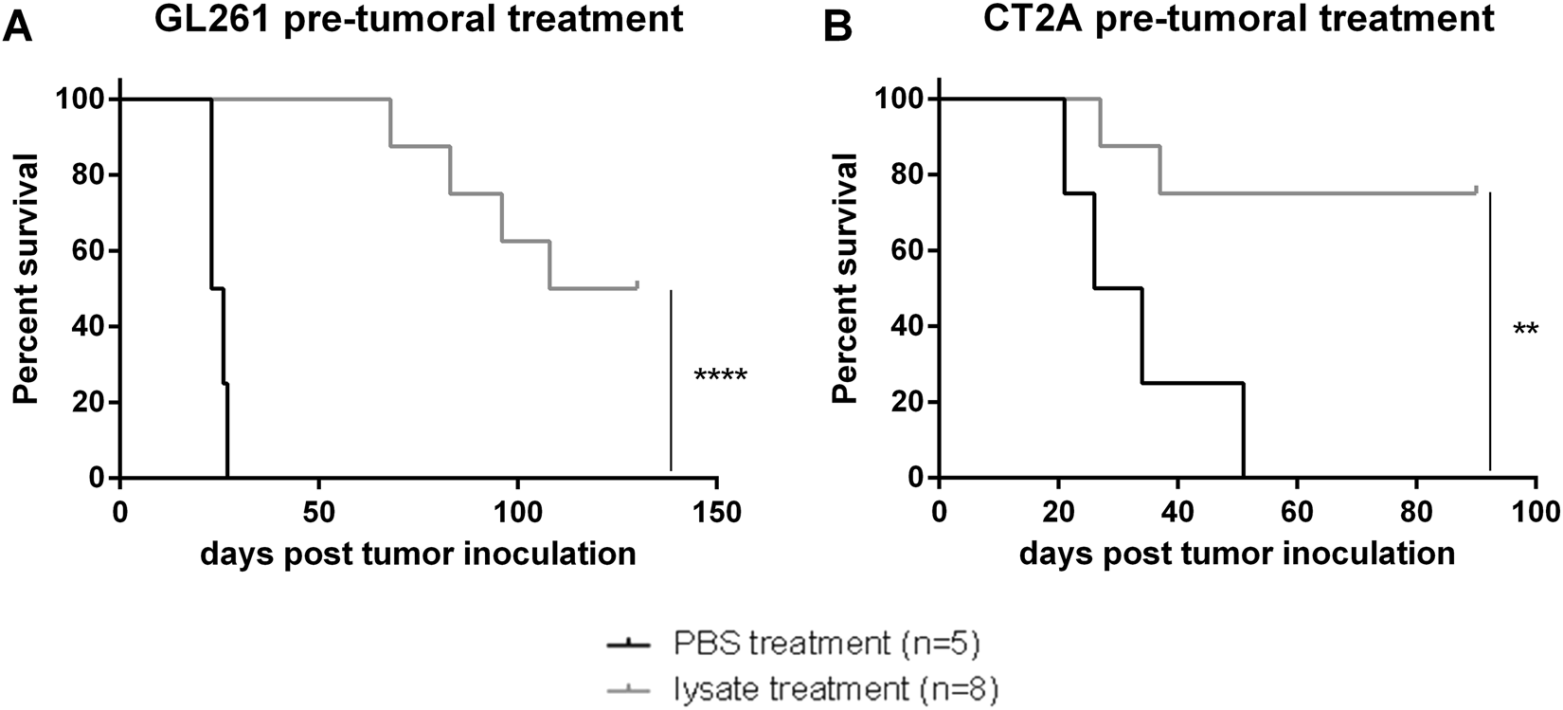
Long-lasting immunological memory induced by pre-tumor lysate treatment. Mice were treated with lysate >100 days prior to tumor inoculation with 5 × 10^5^ glioma cells of the corresponding cell line. In both glioma models, GL261 and CT2A, lysate treated mice were compared to PBS treated control animals. Statistical significance was calculated by Log-rank test, **p < 0.01; ****p < 0.0001.

Another characteristic of the adaptive immunity is the presence of memory cells, which can fasten an immune response in the case of second exposure or relapsed disease. In search for an indication of immunologic memory, mice were pre-treated with lysate more than 100 days prior to tumor inoculation. The experiment was performed in the GL261 and CT2A model and resulted in strong protection from tumor growth (Fig. 4). These results were verified by rechallenge of LT survivors of the experiments described above. The animals were intracranially inoculated with the same amount of autologous tumor cells at the contralateral side of the brain. Nearly 100% survival of the second tumor inoculation was observed in the tested glioma models (Supplementary Fig. 6). Furthermore, specificity of the immune (memory) response was tested by inoculating another group of LT survivors with LLC cells (Supplementary Fig. 6C). All mice developed massive tumor growth, showing specific protection induced by GL261 or CT2A lysate treatment. All of these results demonstrate that pre-tumor lysate treatment gave rise to a tumor-specific memory response, protecting mice for long periods of time and from new tumor inoculation.

### Combining temozolomide and lysate in a curative treatment model improves median survival of glioma-bearing mice

**Figure 5:**
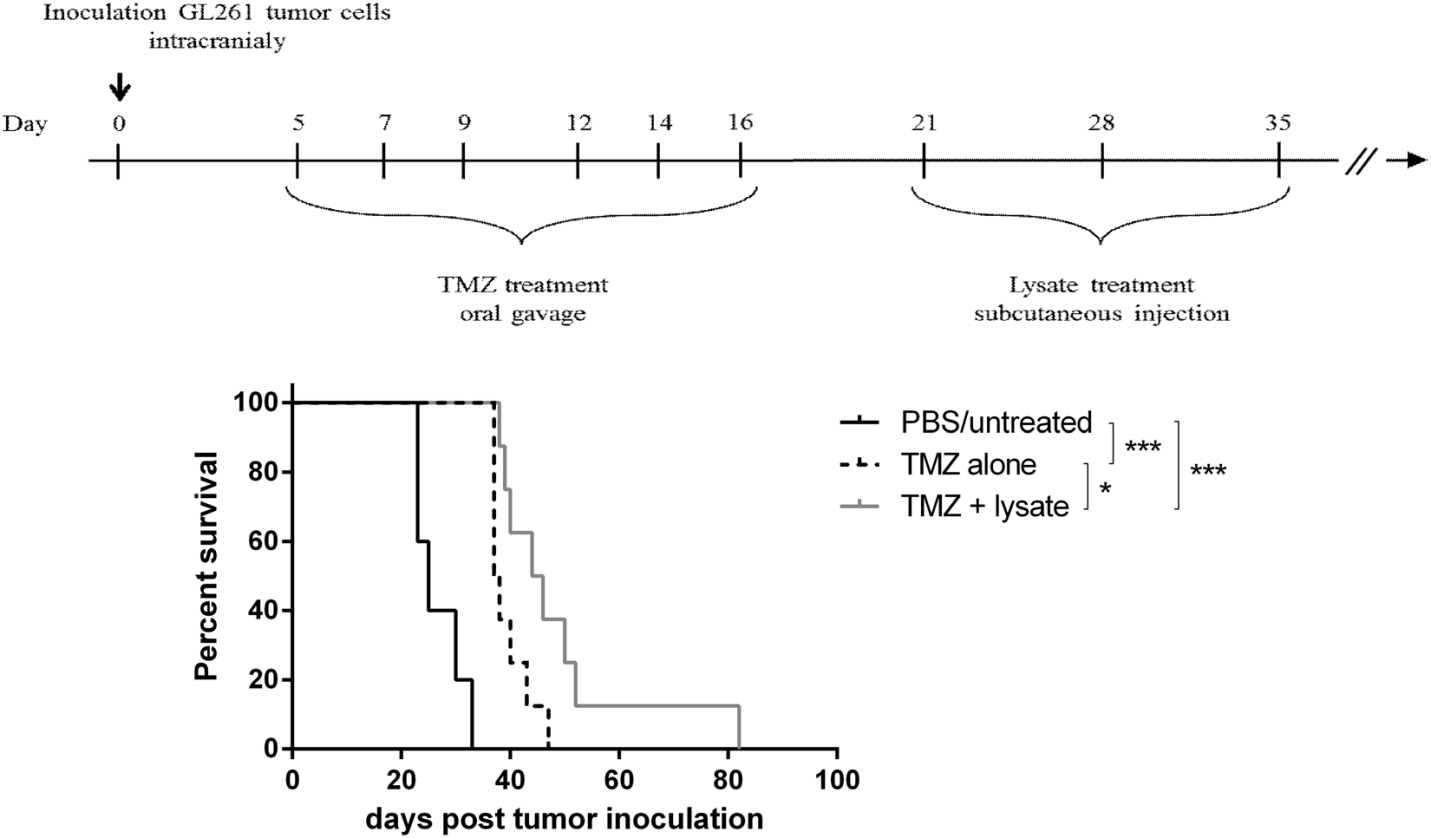
Combining Temozolomide and lysate in a curative treatment improves median survival of glioma-bearing mice. To introduce a curative treatment strategy, lysate injection was combined with chemotherapy (temozolomide). First 20 mg/kg body weight of temozolomide was orally administered 6 times between day 5 and 16, followed by subcutaneous lysate injection at days 21, 28 and 35. The combination of temozolomide and lysate was compared to PBS treated controls and temozolomide monotherapy. One representative experiment out of two; n = 5 in the PBS control group and n = 8 in both test conditions. Statistical significance was calculated by Log-rank test, *p < 0.05; ***p < 0.001.

In a final set of experiments we wanted to test the lysate treatment in a therapeutic setting. Lysate treatment started early after tumor inoculation did not improve survival in comparison to untreated mice (data not shown). Therefore we continued with a more clinical relevant situation in which immunotherapy is performed after direct anti-tumoral therapy such as chemotherapy. For this, a curative treatment strategy was implemented in the GL261 glioma model combining autologous lysate treatment and TMZ. Following tumor inoculation, mice were treated six times with 20 mg/kg bodyweight TMZ between day 5 and 16 and subsequently with autologous lysate injections at day 21, 28 and 35. Similar to data published by Litterman *et al*.^34^, we observed a limited effect of the low TMZ concentration on the presence of T cells and myeloid cell populations in spleen, bone marrow and skin (data not shown). The combination of lysate and TMZ significantly improved the median survival with 80% as compared to mock treated mice and 20% in comparison to TMZ monotherapy (Fig. 5). However, all mice finally died due to tumor growth. The absence of a ‘lysate alone’ condition is due to the fact that most PBS treated mice already show disease symptoms at day 21, which would be the first day to treat the mice in the ‘lysate alone’ group. Moreover, in a preliminary experiment, treatment with lysate alone at days 3, 7 and 11 after tumor inoculation showed no survival benefit (Supplementary Fig. 7). Collectively, immunotherapy using autologous lysate treatment has potential as an adjuvant therapy next to TMZ administration for glioma.

### Subcutaneous, pre-tumor peptide vaccination results in comparable treatment efficacy as compared to peptide loaded DC therapy in the GL261 glioma model

In regard of future perspective, we considered the use of peptide vaccination instead of autologous lysate vaccination. Glycoprotein 100 (gp100), tyrosinase-related protein 2 (TRP-2) and Ephrin A2 (EphA2) are 3 known onco-proteins for GL261 cell line^35,36^. Feasibility of peptide vaccination in the GL261 glioma model was addressed with the CD8 immunodominant epitopes of these three onco-proteins: gp100_25–33_ KVPRNQDWL, TRP-2_180–188_ SVYDFFVWL and EphA2_682–689_ VVSKYKPM (all from lifeTein). Comparing subcutaneous injection of 10 µM peptide mixture (including an equal amount of each peptide) with DCs loaded with this peptide mixture, surprisingly resulted both in a comparable survival benefit (Supplementary Fig. 8). From this it can be concluded that subcutaneous peptide injection, targeting known onco-proteins, can lead to survival benefit even without the addition of *ex vivo* grown DCs.

## Discussion

Within this study, we have shown a positive effect of subcutaneous lysate injections on the survival of glioma-bearing mice. The induced immune response resulted in a brain T cell influx and diminished immune suppression combined with the generation of a tumor specific immunological memory. Moreover, an improved treatment effect of tumor lysate together with TMZ was observed. Based on previous studies within our group, we believe the immunogenicity of the lysate strongly impacts the induction of an immune response^12,13^.

To the best of our knowledge, this is the first preclinical study describing a treatment effect in a murine glioma model by injecting animals with just autologous whole tumor lysate. All publications regarding tumor lysate in the treatment of cancer reside mainly in two groups: the use in DC therapy or in combination with an adjuvant^15–23^. The most striking observation in our study might be the fact that no *ex vivo* grown DCs or adjuvants were needed in this setting. Some authors did report the use of tumor lysate as a negative control condition^15,17,22^. We believe that lysate processed in our hands contains enough activation molecules to load and mature the host’s DCs. Indeed, disrupting the cells via freeze/thawing leads to tumor cell necrosis, which has been shown to cause release of intracellular damage associated molecular patterns (DAMPs) e.g. mitochondrial DNA, uric acid and ATP^37^. Moreover, by using X-ray irradiation after the F/T steps, reactive oxygen species (ROS) are generated^12,38^. These ROS induce protein carbonylation, which increases the immunogenicity of the lysate^12,38^. As the activity of this lysate in DC therapy has already been demonstrated^12^, these observations support the hypothesis of lysate uptake and processing by skin-resident host DCs.

Originally, CD8^+^ T cells were considered the main component of the cellular immune response against cancer^39^. The role of CD4^+^ T cells was presumed to be limited to enhancing and sustaining the CD8^+^ T cell and B cell responses and regulation of the immune response acting as Treg cells^39,40^. More recently CD4^+^ T cells have been pointed out to have more direct roles in cancer immunity with the description of tumor-reactive, cytotoxic CD4^+^ T cells^41–44^. Within our study, the brain T cell influx at day 7 was described to originate from increased proportions of CD4^+^ T cells. Moreover a clear decrease in Treg cells was observed. Although we have no direct proof, CD4^+^ T cells in our model could be involved both in sustaining CD8^+^ T cells as well as having cytotoxic effects. Using a kinetic brain immune-contexture study, focussing on T cells, we were able to detect changes over time with the biggest difference between treated and untreated animals as early as day 7. Although no significant difference in CD8^+^ T cells was detected between lysate treated and mock treated mice at any time point, an increase over time of (activated) CD8^+^ T cells could be observed. We consider this immune influx in the brain of PBS treated mice a tumor response. We hypothesize that the combination of the lower amount of Tregs and activation of CD8^+^ cells later on is able to attack the tumor in lysate treated mice. Importantly, we should mention a bias in our brain immune cell experiment. At day 28, the mock treated group already had two animal deaths whereas the lysate treated group only had one. This might result in slightly skewing of the data as these mice might have had the lowest numbers of (active) T cells in the brain with higher amounts of Tregs.

Based on the presented results, we cannot exclude the contribution of B cells to the immune response in the glioma mouse models. Due to the presence of the blood-brain barrier, antibody permeability is restricted and therefore the humoral response is largely ignored in brain tumor immunotherapy literature^45^. This contrasts with haematological malignancies, in which antibody responses are considered to have an important part in cancer treatment^46^.

In the current study we used two fully immunocompetent glioma mouse models: the standard GL261 model and the CT2A model. Both are syngeneic cell lines for C57BL/6 mice but originate from different cells, the former resembling more of an ependymoblastoma and the latter being more of an anaplastic astrocytoma (together with GBM forming the group of HGG)^30^. The murine GL261 glioma model is the standard model to test immunotherapy in GBM^29^. CT2A cells resemble more the GBM phenotype due to an invasive nature, high mitotic index and cell density^33^. Although the CT2A cell line is stated to be low immunogenic^13^, we were able to show an implementation of an immunotherapeutic treatment modality using lysate treatment. Besides, this is to our knowledge the first report on brain immune cell infiltration in the CT2A model. Moreover, showing one-way cross-reactivity between CT2A and GL261 glioma cell lines can have important implications for paving the way to more universal immunotherapy treatment, as some researchers have suggested the use of multiple HLA-matching reference tumor cell lines to create an universal lysate^18^.

Within our therapeutic treatment experiment we used TMZ treatment, as an anti-tumor therapy, followed by immunotherapeutic autologous lysate injections. Clinical relevance for this treatment combination is provided by Shore *et al*. who describe the positive effect of combining both anti-tumor therapy and immunotherapy in prostate cancer^47^. Starting therapy with the anti-tumor effects of chemotherapy diminishes the tumor burden after which the tumor might recur. Lower tumor burden might also result in a tumor more susceptible to immunotherapy and thus immunotherapy can increase patient survival.

As already stated by our therapeutic treatment experiment as well as in literature, the strength of cancer immunotherapy lies in combining different treatment strategies^48^. In this context, tumor vaccination has already been successfully combined with immune checkpoint blockade, at preclinical level, and oncolytic virotherapy at clinical level. Blocking the negative costimulatory receptor, programmed death 1 (PD-1), showed to be complementary with tumor vaccination in a mouse model of ovarian cancer as well as glioma^49,50^. Within these mice, increased levels of T cell activity, and especially CD8^+^ T cells, were observed while Treg suppression was attenuated. Moreover, immune checkpoint blockade therapies can mediate their effect through reactivation of neo-antigen specific T cells^51^. Likewise, clinical data suggest an anti-tumor effect due to reactivation of existing neo-epitope specific T cells^52,53^. On the other hand, two case reports by Schirrmacher *et al*. describe cancer treatment by combining dendritic cell vaccination and the oncolytic Newcastle disease virus in a patient with prostate cancer and another one with breast cancer^54,55^. In both cases, patients continued a high quality of life after treatment with a long lasting anti-tumor memory T cell response. Taken together, literature shows the importance of combining therapeutic strategies in the treatment of gliomas. Adding autologous lysate treatment might offer similar therapeutic options and next research in our model will evolve in that direction.

Finally, we are aware of a specific drawback of this study as no histopathological sections are investigated. Although flow cytometry indicates attraction of immune cells to the tumor, the possibility exists that they remain in the peri-tumoral environment without infiltrating the tumor itself. However, the study of tumor infiltrating cells results in more quantitative information and is generally accepted^12,13,50,56^. Our data are compatible with T cell activation as demonstrated here by IFN-γ production. A second drawback of our study is that we focus on murine survival which gives no complete insight in the mechanistical events underlying the survival advantage. However, we have clear indication of T cell activation and given the strong differences in murine survival depending on tumor lysate injection, both in pre-tumor implementation and in post-tumor implantation settings (when combined with TMZ), we consider these findings strong enough to find their way into clinical setting.

Taken together all results, autologous lysate treatment can be considered a possible immunotherapy strategy in the treatment of high grade gliomas.

## Conclusion

The present study demonstrated that treatment using autologous whole tumor lysate can dose-dependently initiate an immune response that suppresses tumor growth of orthotopic gliomas. Therefore immunogenic lysate was produced by combining freeze/thawing of tumor cells with high dose gamma irradiation. The effectiveness of lysate treatment for gliomas offers a time and cost-effective approach in comparison to DC therapy, and thus it should be considered as a potential adjuvant treatment for glioma. However, further research is required to refine this treatment strategy and to improve therapeutic effects on gliomas. In respect to optimisation, nanoparticles form an attractive path combining lysate delivery and adjuvant properties.

## Methods

### Glioma cell lines and cell culture

GL261 glioma cells were kindly provided by Dr. Ilker Eyüpoglu from the University of Erlangen (Germany). CT2A cells were a generous gift from Prof. Holger Gerhardt from the Vesalius Research Center (VIB, Leuven, Belgium) ﻿﻿with MTA obtained from Prof. Thomas N. Seyfried (Boston College, USA)﻿. Both C57BL/6 syngeneic tumor cell cultures were maintained at 37 °C under 5% CO_2_ in DMEM supplemented with 10% heat-inactivated fetal calf serum (FCS), 100 U/ml penicillin, 100 µg/ml streptomycin (pen/strep) and 2 mM L-glutamine (L-Glu) (all from Lonza, Verviers, Belgium).

### Tumor lysate generation and FITC labeling

Starting with a concentration of 40 × 10^6^ tumor cells per ml 1% PBS/FCS, lysate was created by performing 6 freeze-thaw (F/T) cycles consisting of 3 minutes liquid nitrogen and 3 minutes 56 °C warm water bath. Afterwards the lysate was irradiated (IRR) with 60 Gy to increase immunogenicity^12^. Protein concentration was determined by Bradford protein assay (Bio-Rad, Temse, Belgium). Tumor cell avitality was checked with trypan blue dye exclusion.

In some experiments, lysate was labelled with fluorescein isothiocyanate (FITC) for the detection by microscopy and flow cytometry^27^. In short, 20 μl FITC (Sigma-Aldrich, Diegem, Belgium) dissolved in 5 mg/ml dimethyl sulfoxide (DMSO, WAK-chemie Medical, Germany) was added per mg protein in labeling buffer containing 0.05 M boric acid and 0.2 M NaCl at pH 9.2 using a 10.000 molecular weight cut-off amicon ultra 15 centrifugal filter (Merck, Overijse, Belgium). After 2 h incubation at room temperature, unbound FITC was removed by gel filtration using an Econo-Pac® 10DG Column (Bio-Rad). Finally, the protein concentration was measured with a SmartSpec Plus Spectrophotometer (Bio-Rad) at 280 nm and 492 nm.

### DC generation and culturing

Bone marrow derived DCs were obtained by isolation of femur and tibia from 8 to 10 weeks old female C57BL/6 J mice (Envigo, Horst, The Netherlands). Bone marrow was flushed using PBS followed by erythrocyte lysis. Finally, progenitor cells were counted (Micros 60, Horiba ABX, France) and cultured at 10^6^ cells per ml DC-medium consisting of RPMI-1640 medium (Lonza) supplemented with 10% FCS, pen/strep, L-Glu, 50 µM β-mercaptoethanol (Sigma-Aldrich) and 20 ng/ml granulocyte-macrophage colony-stimulating factor (GM-CSF, Peprotech, US). At days 3 and 5 medium was refreshed and at days 6 and 7 the cells were prepared for flow cytometry or microscopy. On day 7 iDCs can be collected to develop mature DCs using 100 µg glioma lysate per 10^6^ cells in the above medium supplemented with 1 µg/ml *Escherichia coli* lipopolysaccharide (LPS, Sigma-Aldrich). To check for maturation, maturation markers of DCs were stained (Table 1). Flow cytometry was performed using a LSR Fortessa Analyzer (BD Biosciences, Erembodegem, Belgium) and analysed using FlowJo software (Tree Star, Ashland, OR, US).

**Table 1:** Antibodies for flow cytometry.

Antigen	Fluorochrome	Company
**Surface stainings – DC maturation**		
CD11c	APC	eBioscience
CD80	PE	eBioscience
CD86	PE	BD
CD40	PE	BD
H2Kb (MHC-I)	PE	BD
IA/IE (MHC-II)	PE	BD
**Surface stainings – Brain immune cells**		
CD45	AF700	eBioscience
CD3	FITC/PE	eBioscience
CD4	PerCP Cy5.5/eF780	eBioscience
CD8	BV421	BD
NKp46	APC	Biolegend
**Intracellular stainings – Brain immune cells**		
Foxp3	PE	eBioscience
IFN-γ	PerCP Cy5.5	BD
Viability marker	Zombie Yellow	Biolegend

### Analysis of lysate uptake by dendritic cells and presentation in MHC class I molecules

Immature DCs (iDCs) at day six of culture were seeded on coverslips for 24 hours and incubated with FITC-labelled lysate for 90 minutes. Afterwards, cells were washed extensively, followed by fixation and nucleus staining with DAPI (Sigma-Aldrich). To study uptake, surface FITC quenching with trypan blue was conducted using 10 min incubation in 0.1% (w/v) trypan blue solution^57^. In order to show antigen presentation by major histocompatibility complex class I (MHC-I) molecules, cells were washed after the 90 min incubation and matured during 24 hours. Cells were stained with PE-anti-MHC-I for 30 minutes before fixation and nucleus staining^27^. Finally, the coverslips were placed on microscope slides and an Olympus BX41 Fluorescence Microscope (US) and LSM 510 meta confocal microscope (Zeiss, Germany) were used to examine the samples.

### Orthotopic glioma mouse models

Female C57BL/6 J mice were treated prior to (pre-tumor treatment) or shortly after (therapeutic treatment) tumor inoculation. Unless stated otherwise, all experiments were performed with 100 µg lysate of a 1 mg/ml concentration. For all *in vivo* experiments freshly prepared lysate was used and all injections were administered in a total volume of 100 µl. All animal experiments were approved by the bioethics committee of the KU Leuven and conducted according to international guidelines.

#### Pre-tumor treatment

Mice of 8 to 10 weeks old were intraperitoneally (i.p.) or subcutaneously (s.c.) (in the abdominal wall) injected 14 and 7 days before tumor inoculation. Treatment modalities consisted of PBS-treated (mock) controls and lysate treatment. At day 0, mice were intracranially injected with 5 × 10^5^ glioma cells in a volume of 10 µl^31^. Briefly, mice were anaesthetized and fixed in a stereotactic frame. Tumor cells were injected 2 mm posterior and lateral from the bregma at 3 mm depth. Afterwards, follow-up of the mice was performed two to five times a week by weight and scoring based on a neurological deficit scale adapted by Maes *et al*.^31^. Mice surviving longer than three times the median survival of mock treated animals were considered long-term (LT) survivors. Without any additional treatment, tumor inoculation was repeated in these animals at the contralateral hemisphere. Naïve mice, older than 20 weeks, were used as age-matched control condition. In another set of experiments, tumor specificity was tested using a Lewis lung carcinoma (LLC) cell line, obtained from Prof. Patrizia Agostinis (KU Leuven, Belgium). Therefore LT surviving animals and age-matched controls were inoculated subcutaneously with LLC cells or intracranially with glioma cells.

#### Therapeutic treatment

In these survival experiments lysate treatment was combined with temozolomide (TMZ) administration. Tumor inoculation was performed as described above and TMZ was prepared as described previously^13^. In brief, the content of Temodal® capsules (MSD, Hertfordshire, UK) together with an equal amount of L-Histidine was dissolved in a phosphate buffer and administered in a total volume of 200 µl by oral gavage (Schering-Plough, Belgium). Prior to treatment, mice were randomised over the different groups. Mice received 20 mg/kg TMZ at days 5, 7, 9, 12, 14, 16 and subcutaneous lysate injections at day 21, 28 and 35.

### Brain immune cell isolation and characterisation

Mononuclear cells in the brain of pre-treated, CT2A-inoculated mice were isolated 7, 14, 21 or 28 days post tumor inoculation as previously described^31^. Briefly, isolated brains were digested in Collagenase D (Roche, Anderlecht, Belgium) and DNase (Invitrogen), and filtered prior to Percoll gradient centrifugation (Sigma). After recovering the mononuclear cell interphase, assessment of the immune cells was done by flow cytometry. Therefore surface staining for T cell markers was performed following zombie yellow viability staining (Table 1). Foxp3-PE staining kit (eBioscience, Vienna, Austria) was used according to the manufacturer’s protocol to detect intracellular Foxp3. For the intracellular IFN-γ staining, cells were stimulated for 3 hours *in vitro* with 100 ng/mL phorbol myristate acetate, 1 mg/mL ionomycin and 0.7 mg/mL monensin (all from Sigma-Aldrich). After viability and surface staining cells were permeabilized with a buffer containing 0.5% saponin and 0.5% bovine serum albumin prior to IFN-γ staining.

### Statistical analysis

Two-way analysis of variance (ANOVA) testing was used for comparing multiple groups. Data are represented as median. Survival analyses were performed using the Log-rank (Mantel-Cox) test. All data were analysed using Graphpad Prism software 6 (Graphpad Software, San Diego, USA) and statistical significance was considered if p < 0.05.

### Data Availability

The datasets generated during and/or analysed during the current study are available from the corresponding author on reasonable request.

## Electronic supplementary material

Supplementary Information

Supplementary video
