# Supplementary material for: Immunotherapy with subcutaneous immunogenic autologous tumor lysate increases murine glioblastoma survival
Source: Sci Rep. 2017 Oct 24;7:13902. doi: 10.1038/s41598-017-12584-0 (PMC5654749; doi:10.1038/s41598-017-12584-0)
Supplement: Supplementary file 1 — Supplementary Information [file 41598_2017_12584_MOESM1_ESM.pdf]

Immunotherapy with subcutaneous immunogenic autologous tumor lysate increases murine glioblastoma survival

Jochen Belmans(1), Matthias Van Woensel(2,3), Brecht Creyns(4), Joost Dejaegher(2), Dominique M. Bullens*,#(1,5), Stefaan W. Van Gool# (1,6)

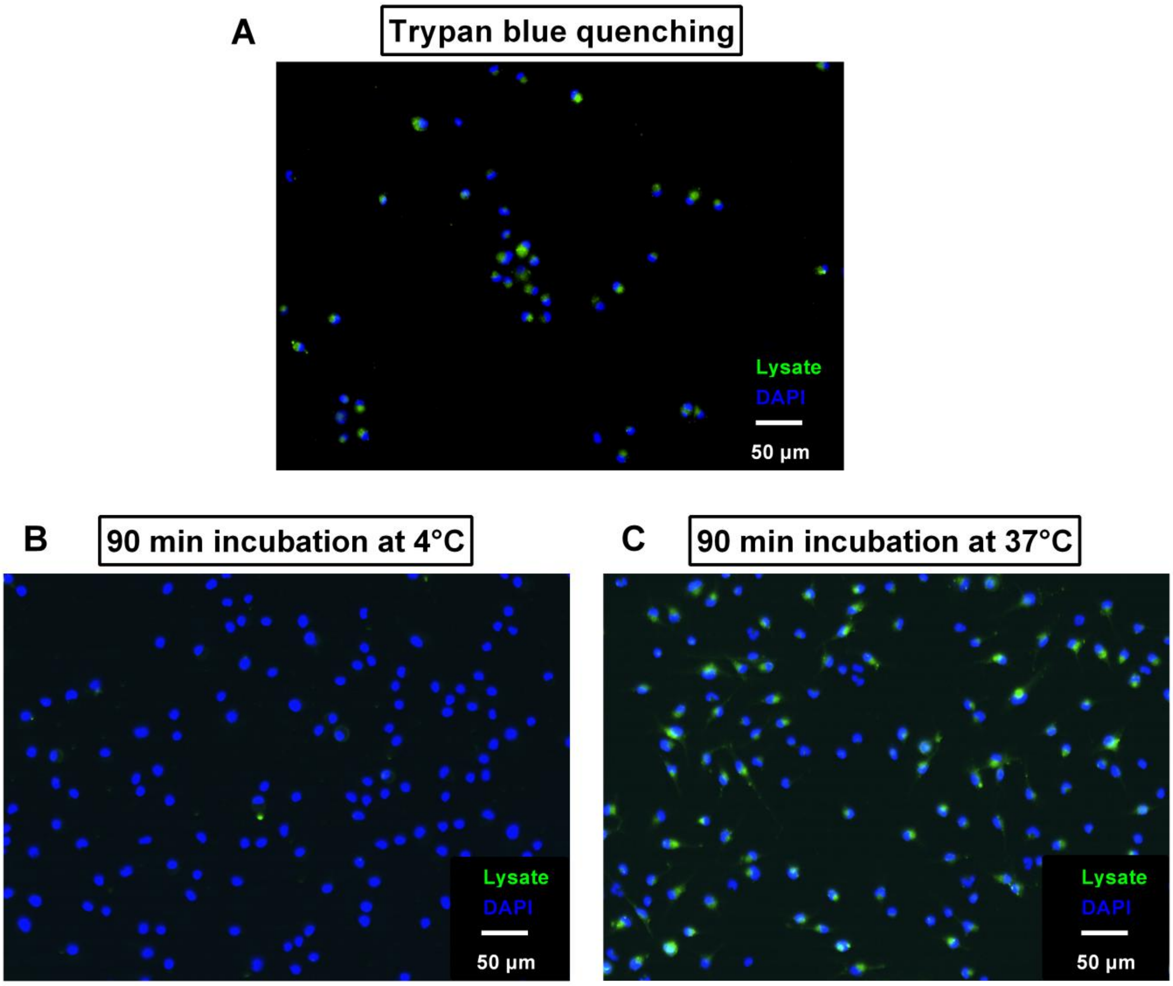

Supplementary Figure 1: Uptake of lysate fragments by DCs is an active process. *In vitro* differentiated DCs were incubated with FITC labelled lysate for 90 minutes, afterwards washed and stained. **(A)** Trypan blue quenching of surface FITC signal (green) was used to show uptake of lysate fragments by DCs. **(B),(C)** To show uptake is an active process, incubation at 4°C was performed in comparison to 37°C. At 4°C no active, ATP-requiring processes take place. Images were visualized with a 200x magnification and are representative of multiple independent experiments. DAPI nucleus staining (blue) and FITC signal (green)

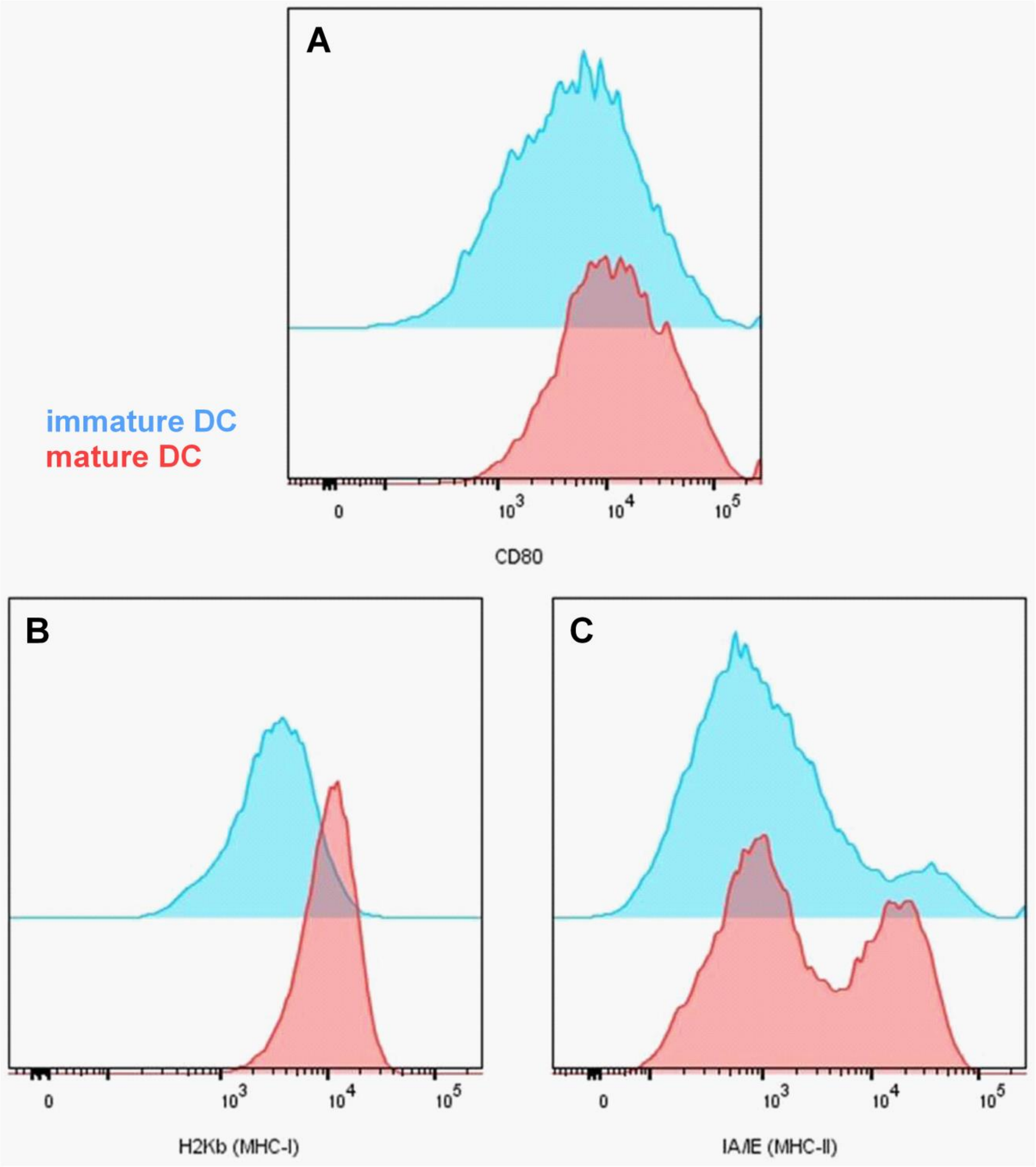

Supplementary Figure 2: Increased expression of maturation markers by lysate-loaded dendritic cells. *In vitro* differentiated DCs were incubated with lysate for 90 minutes, afterwards washed and incubated for 24 hours. Flow cytometric staining was used to study the expression of maturation markers on lysate loaded DCs. Representative graphs for DC maturation markers **(A)** CD80, **(B)** IA/IE (MHC-II molecule) and **(C)** H2Kb (MHC-I molecule) are shown with expression on immature DCs in blue and marker expression by mature DCs in red. Graphs are representative of at least three independent experiments.

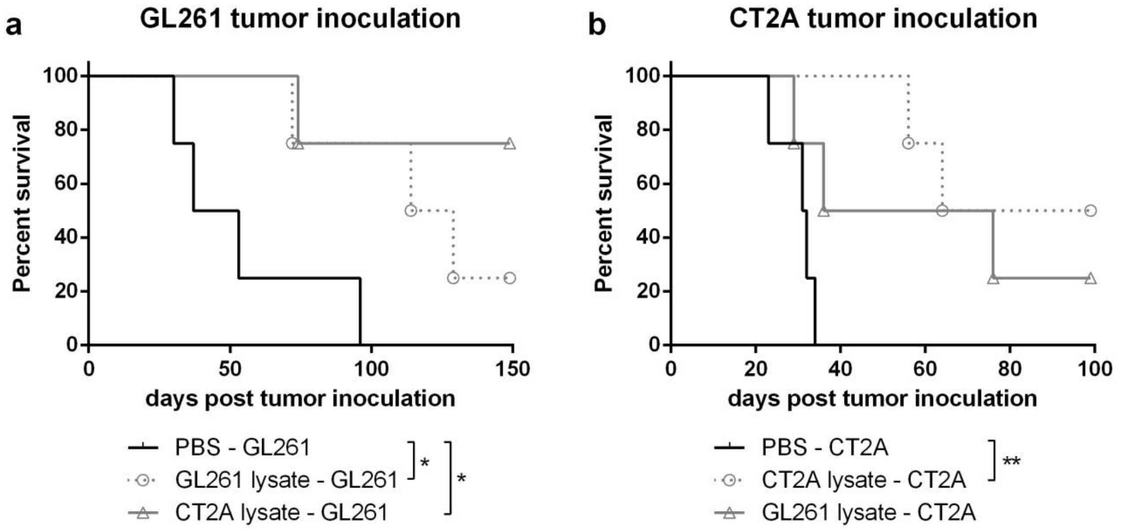

Supplementary Figure 3: Lysate of CT2A glioma cells is effective in treating mice with an orthotopic tumor inoculation of GL261 glioma cells. Both GL261 and CT2A cell lines are syngeneic cell lines for C57Bl/6J mice. Using one tumor cell line to pre-treat and tumor inoculation with the other cell line, cross-reactivity between both cell lines was investigated. For all conditions four mice per group were used. Statistical significance was calculated by Log-rank test, * $p < 0.05$; ** $p < 0.01$.

Gating strategy - FoxP3

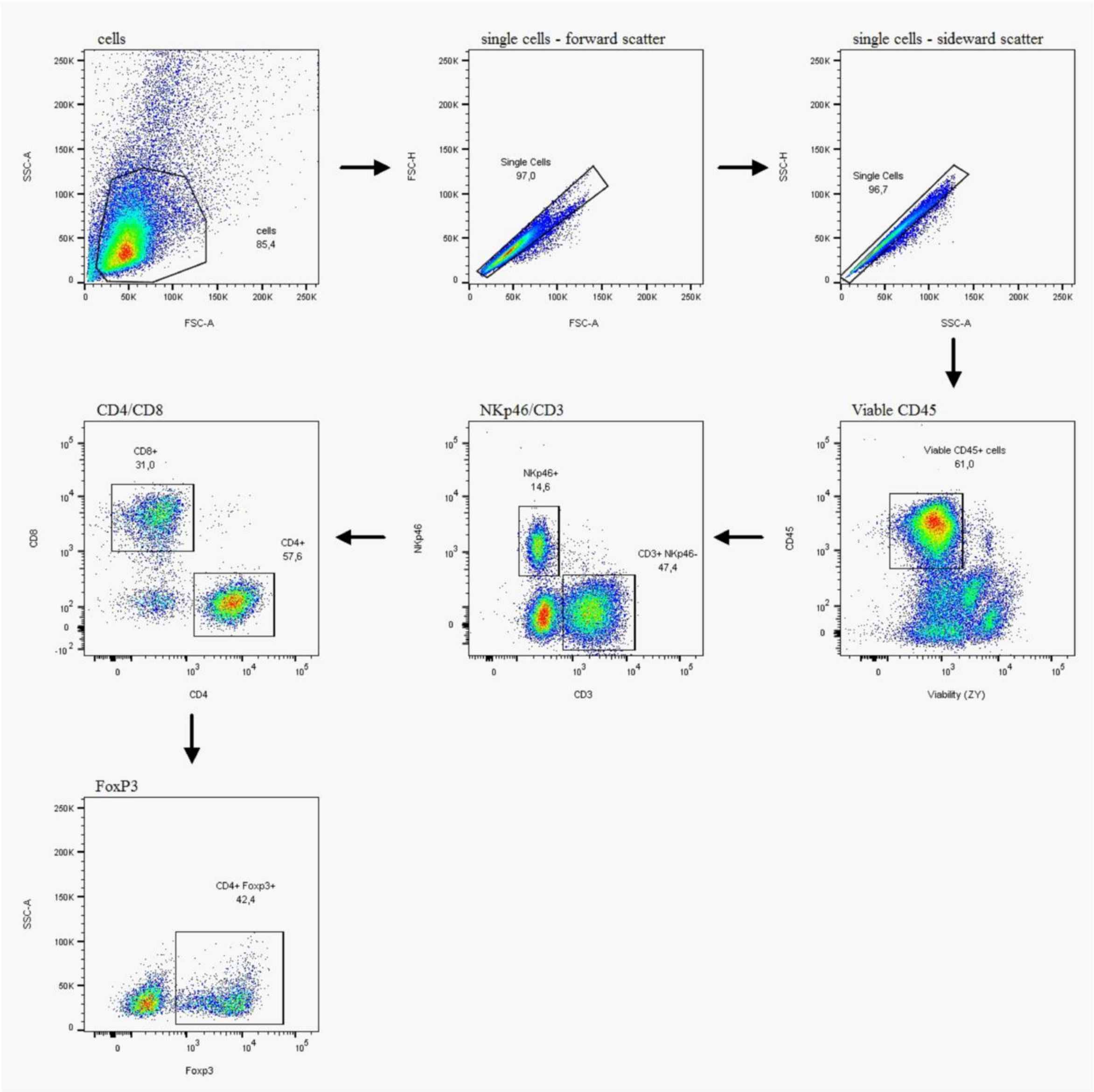

Supplementary Figure 4: Gating strategy for flow cytometry of brain regulatory T cell influx. Flow cytometry was performed on brain infiltrating cells of pre-treated mice and gated for cells, to remove debris, single cells (via SSC and FSC), and viable leukocytes (CD45+ and ZY-). Next, the $CD3^{+}NKp46^{-}$ lymphocytes were selected and further subdivided in $CD4^{+}$ and $CD8^{+}$ T cells. Finally, the expression of FoxP3 within the $CD4^{+}$ gate was monitored.

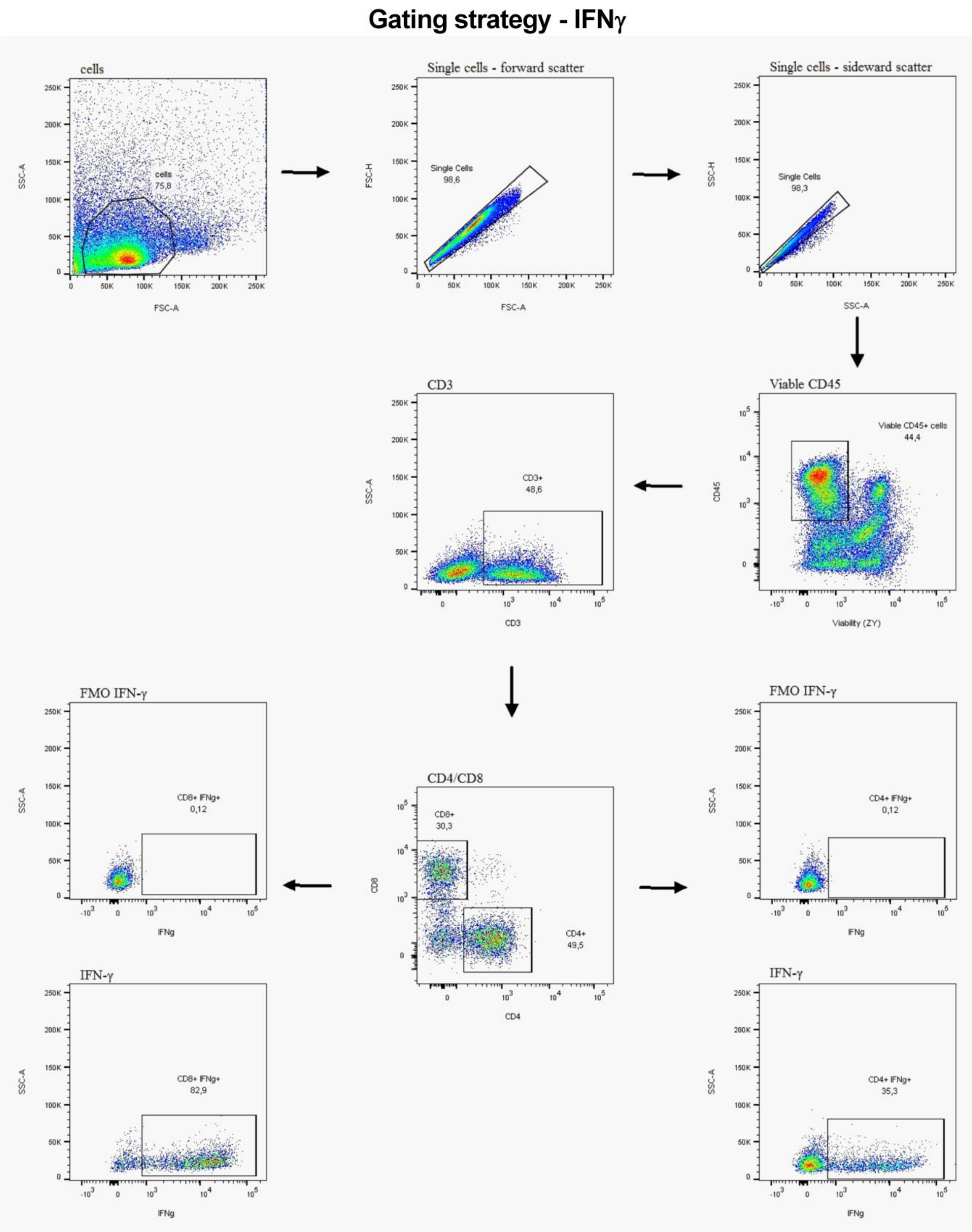

Supplementary Figure 5: Flow cytometric gating strategy of IFN-γ producing brain immune cells. Flow cytometry was performed on isolated mononuclear brain infiltrating cells of pre-treated mice and gated for cells (to remove debris), single cells (via SSC and FSC), viable leukocytes (CD45+ ZY-), and CD3^{+} lymphocytes. Subsequently, we looked into IFN-γ production of CD4 and CD8 T cells. Therefore we used FMO (fluorescence minus one) to determine the proper IFN-γ gating strategy.

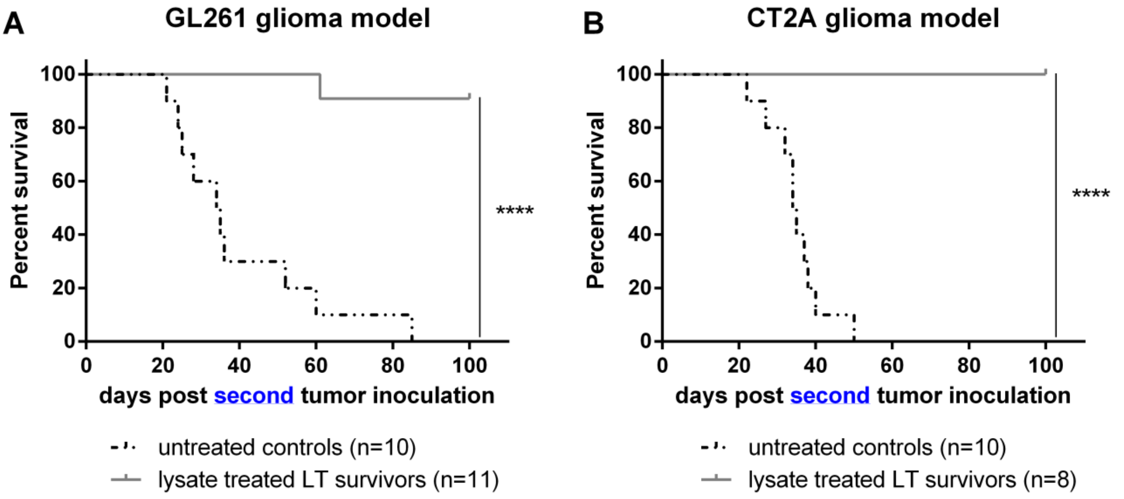

C

Tumor incidence in long term survivors and age-matched controls implanted intracranially with glioma cells, or subcutaneously with LLC cells		
	Tumor inoculation age-matched controls	Rechallenge of long term-survivors
GL261	10/10	1/10
LLC	5/5	6/6
CT2A	10/10	0/10
LLC	4/4	3/3

Supplementary Figure 6. Tumor-specific immunological memory protects long term surviving mice from second tumor inoculation. Mice that survived the first tumor inoculation were rechallenged at the contralateral side of the brain with 5 x 10^5 glioma cells of the same tumor cell line as the first time without new treatment. New untreated controls were used as a control condition. **(C)** Tumor specificity was tested by inoculating other groups of long term survivors with LLC cells subcutaneously. In graphs **(A)** and **(B)** data of two independent experiments were pooled. Statistical significance was calculated by Log-rank test, **** $p < 0.0001$.

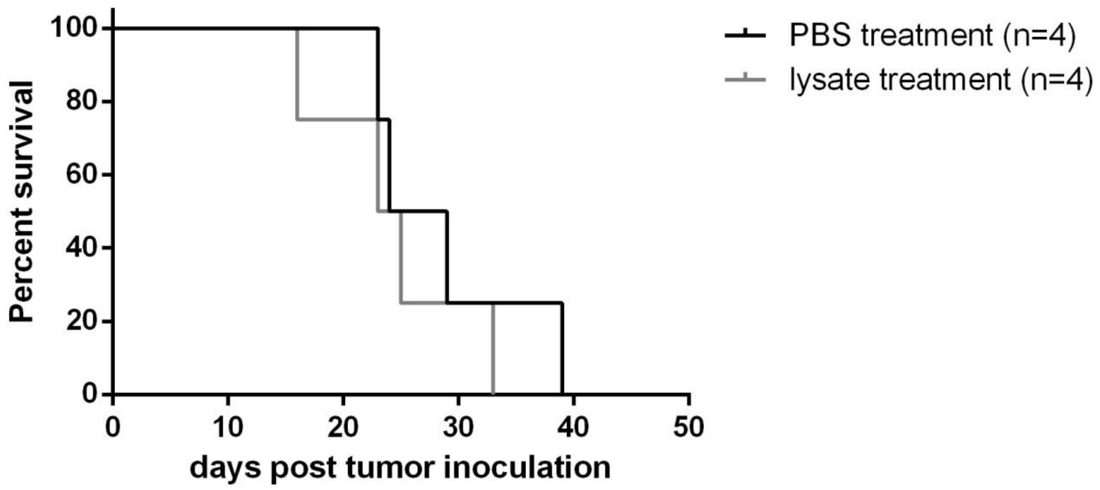

Supplementary Figure 7: Standalone therapeutic lysate treatment did not result in a survival advantage for glioma bearing mice. After tumor inoculation with 5 x 10^5 GL261 cells, mice were subcutaneously treated with autologous lysate at days 3, 7 and 11. Injection of lysate was compared to PBS treatment.

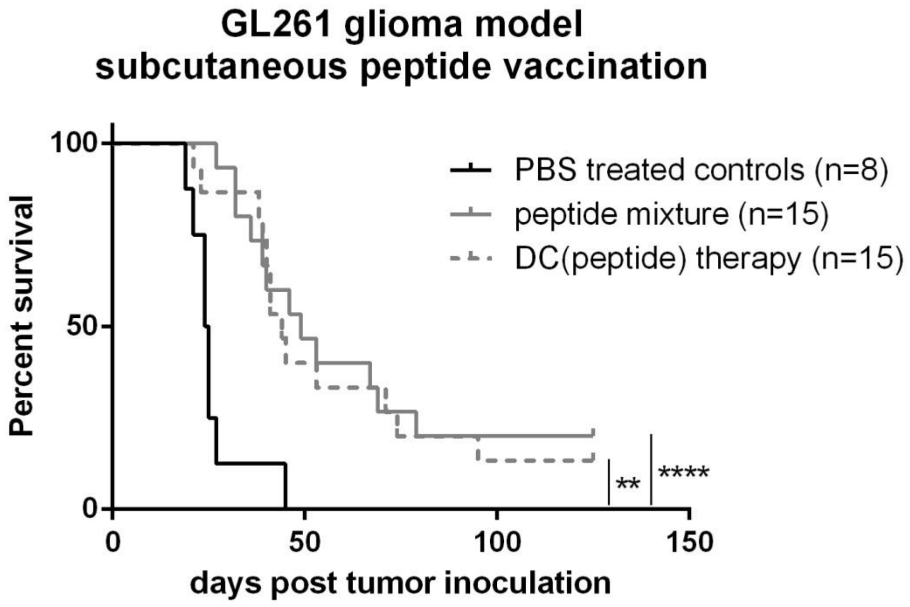

Supplementary Figure 8: Subcutaneous, pre-tumor peptide vaccination results in comparable treatment efficacy as compared to peptide loaded DC therapy in the GL261 glioma model. Mice were treated 14 and 7 days before tumor inoculation with 5 x 10^5 GL261 cells. Treatment consisted of a mixture of the CD8 immunodominant peptides of 3 different onco-proteins (gp100, TRP-2 and EphA2) injected subcutaneously as such or when loaded to DCs. Statistical significance was calculated by Log-rank test, ** $p < 0.01$; $p < 0.0001$

Supplementary Video: Uptake of lysate fragments by dendritic cells *in vitro*. *In vitro* differentiated DCs were incubated with FITC labelled lysate for 90 minutes, afterwards washed and stained. Z-stack images, obtained with confocal microscopy, were used to visualize DCs and prove real uptake of lysate. Nine slices of the confocal z-stack with DAPI nucleus staining (blue) and FITC signal (green) were merged to a video moving through the cells from bottom to top. Images were visualized with a 400x magnification.
